# Impairment of respiratory muscle strength in Berardinelli-Seip congenital lipodystrophy subjects

**DOI:** 10.1186/s12931-018-0879-8

**Published:** 2018-09-12

**Authors:** Jorge Luiz Dantas de Medeiros, Bruno Carneiro Bezerra, Thiago Anderson Brito de Araújo, Aquiles Sales Craveiro Sarmento, Lázaro Batista de Azevedo Medeiros, Lucien Peroni Gualdi, Maria do Socorro Luna Cruz, Thaiza Teixeira Xavier Nobre, Josivan Gomes Lima, Julliane Tamara Araújo de Melo Campos

**Affiliations:** 1 Faculdade de Ciências da Saúde do Trairi, Universidade Federal do Rio Grande do Norte, Santa Cruz, RN Brazil; 2 Laboratório de Biologia Molecular e Genômica, Departamento de Biologia Celular e Genética, Centro de Biociências, Universidade Federal do Rio Grande do Norte, Natal, RN Brazil; 3 Departamento de Medicina Clínica, Hospital Universitário Onofre Lopes (HUOL)/UFRN, Natal, RN Brazil

**Keywords:** Lipodystrophy, MIP, MEP, PMS, Metreleptin

## Abstract

**Background:**

Berardinelli-Seip Congenital Generalized Lipodystrophy (BSCL) is an ultra-rare metabolic disease characterized by hypertriglyceridemia, hyperinsulinemia, hyperglycemia, hypoleptinemia, and diabetes mellitus. Although cardiovascular disturbances have been observed in BSCL patients, there are no studies regarding the Respiratory Muscle Strength (RMS) in this type of lipodystrophy. This study aimed to evaluate RMS in BSCL subjects compared with healthy subjects.

**Methods:**

Eleven individuals with BSCL and 11 healthy subjects matched for age and gender were included in this study. The Maximum Inspiratory Pressure (MIP), Maximum Expiratory Pressure (MEP), and Peripheral Muscle Strength (PMS) were measured for three consecutive years. BSCL subjects were compared to healthy individuals for MIP, MEP, and PMS. Correlations between PMS and MIP were also analyzed. The genetic diagnosis was performed, and sociodemographic and anthropometric data were also collected.

**Results:**

BSCL subjects showed significantly lower values for MIP and MEP (*p* <  0.0001 and *p* = 0.0002, respectively) in comparison to healthy subjects, but no changes in handgrip strength (*p* = 0.15). Additionally, we did not observe changes in MIP, MEP, and PMS two years after the first analysis, showing maintenance of respiratory dysfunction in BSCL subjects (*p* = 0.05; *p* = 0.45; *p* = 0.99). PMS and MIP were not correlated in these subjects (*r* = 0.56; *p* = 0.18).

**Conclusion:**

BSCL subjects showed lower respiratory muscle strength when compared with healthy subjects; however, PMS was not altered. These findings were maintained at similar levels during the two years of evaluation. Our data reveal the first association of BSCL with the development of respiratory muscle weakness.

**Electronic supplementary material:**

The online version of this article (10.1186/s12931-018-0879-8) contains supplementary material, which is available to authorized users.

## Background

Berardinelli-Seip Congenital Lipodystrophy (BSCL) is a rare genetic disease characterized by the absence of adipose tissue that contributes to the impairment of glucose and lipid metabolism [1, 2]. At the metabolic level, BSCL patients present dyslipidemia, hyperinsulinemia, disrupted carbohydrate metabolism, insulin resistance, diabetes mellitus (DM), hepatosplenomegaly, hepatic steatosis, acanthosis nigricans, low levels of leptin and adiponectin, decreased levels of high-density lipoprotein (HDL), and hypertriglyceridemia [2, 3]. The phenotype of BSCL patients is typical, revealing acromegaloid facies, prognathism, prominent musculature, hypertrophic cardiomyopathy, umbilical protrusion, acanthosis nigricans, and other clinical features [1–5].

Worldwide prevalence of BSCL has been estimated in 1:10,000,000 live births, with approximately 500 cases around the world [6, 7]. In Brazil, a high prevalence of BSCL was found in the Rio Grande do Norte (RN) state, in the Northeast region (3 in 100,000), with 103 cases described [7]. Clinical, laboratory and genetic data for BSCL patients from RN were reported by Lima et al. [3]. Genetic data were also reported by Medeiros et al. [7]. Recently, our research group analyzed the leading causes of death in BSCL patients from RN. We found that many of our patients died due to infections, mainly pulmonary [8].

It is well known that maximal respiratory pressure impairment leads to ventilatory inefficiency. Besides the ventilatory importance, inspiratory and expiratory muscles are also important in maintaining upper airway patency [9]. Studies have shown that several chronic conditions may lead to respiratory muscle weakness (RMW). Inspiratory muscle strength is decreased in 30–50% of patients with chronic heart failure [10, 11], and is also associated with autonomic cardiovascular dysfunction in type 2 diabetic patients [12]. Additionally, RMW was also found in type 2 diabetic patients [13]. Considering the metabolic, cardiovascular, and cognitive complications related to BSCL subjects worldwide [5, 14–16], the abnormal cardiovascular autonomic modulation shown by BSCL subjects from RN [4, 5], as well as the lack of studies investigating respiratory maximal pressures in these subjects, the aim of this research was to evaluate the respiratory and peripheric muscle strength in BSCL subjects compared to healthy subjects.

## Methods

### Study design and data collection

This cross-sectional and longitudinal study was conducted from November 2015 to December 2017. The size sample was not precisely calculated. Eleven BSCL subjects from Seridó of RN were recruited in 2015 by *ASPOSBERN* (*Associação de Pais e Pessoas com a Síndrome de Berardinelli do Estado do Rio Grande do Norte*), a non-profit organization aiming to improve the life quality of BSCL subjects and their families. This organization performs an important role in the management of BSCL subjects diagnosed by qualified physicians and researchers from the Brazilian Research Group for Studies about the Genetics and Morphophysiological Features of Berardinelli-Seip Lipodystrophy [7]. Four BSCL individuals of our study were lost to follow-up: one had died (subject 9) and three did not participate in the 2016 and 2017 data collection (subjects 2, 6, and 11). However, the participant number was considered high since BSCL is an ultra-rare disease with approximately 500 cases described worldwide [3] and the RN state has the highest prevalence documented for this disease [7]. Subjects included in the study participated in a one-day standard physical therapy program during the Annual Meeting of ASPOSBERN held in 2015 to 2017. Inclusion criteria were to be genetically and/or clinically diagnosed with BSCL, to be older than 18 years-old, to be able to understand the tests, and to sign the Written Informed Consent Form (WICF). We excluded subjects who had previously had respiratory diseases or who did not understand how to perform the test. Healthy individuals matched for age and gender were recruited in the Clínica Escola de Fisioterapia, da Faculdade de Ciências da Saúde do Trairi, campus da Universidade Federal do Rio Grande do Norte (UFRN), during February 2016. Inclusion and exclusion criteria were the same as those for BSCL subjects, except for BSCL diagnosis. Table 1 summarizes the clinical, metabolic, and genetic features of BSCL subjects. These data for all BSCL patients from RN were previously described by Lima et al. [3] and Medeiros et al. [7]. Sociodemographic and anthropometrical data were also collected. Participants were also asked about the time after BSCL diagnosis and their practice and weekly frequency of physical activities, according to the International Physical Activity Questionnaire (IPAQ), by using the 8-question short version [17–19]. Table 3 summarizes the physical activity profile of BSCL subjects among the three annual evaluations. Some BSCL patients started the use of metreleptin replacement to treat complications of leptin deficiency, such as insulin resistance, diabetes, and/or hypertriglyceridemia. The metreleptin replacement therapy was performed according to Musso and co-workers [20].

**Table 1:** Clinical and genetic data of BSCL subjects

Case	Gender/Age (Years)	Comorbidities	Mutated gene	Drugs	Metreleptin use (since year)	RMW (Yes/No)	PMW (Yes/No)
1	♀ / 21	DM, HT, SH	*AGPAT2*	ISL	*Yes (2016)*	*Yes*	No
2^a^	♀ / 22	DM, HT, HT, SH	*BSCL2*	ISL, RMP	–	*Yes*	No
3	♂ / 24	DM, HT	*BSCL2*	MTF, HCT, SXG, CPF, RMP	*Yes (2016)*	*Yes*	No
4	♀ / 28	DM, AH, HT, SH	*BSCL2*	ISL	*Yes (2017)*	*Yes*	No
5	♂ / 29	DM, AH, HT, SH	*BSCL2*	ISL, SXG, MTF, RMP	*Yes (2016)*	*Yes*	No
6^a^	♀ / 29	DM, HT, SH	*BSCL2*	ISL	*–*	*Yes*	No
7	♂ / 31	DM, HT	*BSCL2*	MTF, SVT	–	Yes	No
8^b^	♂ / 36	DM, AH, HT	*BSCL2*	NA	*–*	*Yes*	No
9^a^	♀ / 39	DM, AH, HT, KF	*BSCL2*	ISL	*–*	*Yes*	No
10	♀ / 43	DM, HT	*BSCL2*	ISL	*–*	*Yes*	No
11^a^	♀ / 44	DM, AH, HT	*BSCL2*	ISL, MTF, ATN, HCT	–	Yes	No

*DM* diabetes mellitus, *AH* arterial hypertension, *HT* hypertriglyceridemia, *SH* steatohepatitis, *KF* kidney failure, *RMW* Respiratory Muscle Weakness, *MTF* Metformin, *HCT* Hydrochlorothiazide, *SXG* Saxagliptin, *CPF* Ciprofibrate, *RMP* Ramipril, *ISL* Insulin, *SVT* Sinvastatin, *ATN* Atenolol

^a^Subjects that participated in 2015 but were lost to follow-up in 2016–2017. ^b^BSCL subject that declared to be a smoker

### Biochemical measurements in plasma

The measurement of glucose, triglycerides and total cholesterol was performed following the instructions from the Labtest protocols (Lagoa Santa, Brazil) and LABMAX PLENNO equipment (LABTEST, Lagoa Santa, Brazil) [21]. Glucose was determined by end-point method of glucose oxidase and with the measurement of absorbance in 505 nm. Triglycerides was determined by enzymatic method by end-point and with the measurement of absorbance in 505 nm. Total cholesterol was also determined by enzymatic method by end-point and with the measurement of absorbance in 500 nm.

### Maximum respiratory pressure measurement

We assessed respiratory muscle strength by measuring Maximum Inspiratory Pressure (MIP) and Maximum Expiratory Pressure (MEP) using an analogic manuvacuometer (MV 300®, WIKA +/− 300 cmH_2_O) attached to an unidirectional valve connected to a paper mouthpiece. Subjects were in sitting position with a nose clip in place to prevent nasal air leakage. The MIP and MEP pressures were measured according to *American Thoracic Society* and *European Respiratory Society* (ATS/ERS) [22]. The maximal pressure was maintained for at least 1 second, and 3 to 5 maneuvers were performed [23]. Reference values standardized for the studied population were used [24].

We evaluated both respiratory pressures at three time-points: 2015, 2016, and 2017. The respiratory muscle weakness (RMW) state was defined as having MIP and/or MEP values lower than 70% of predictive values, as previously described. Therefore, healthy subjects with predicted values lower than 70% were considered unsuitable and, therefore, were excluded [25–27]. Results are shown in percentage of predicted (%) values.

### Peripheral muscle strength measurement

Peripheral muscle strength was measured by handgrip dynamometry using a hydraulic dynamometer (Saehan Corporation), which measures the strength of a grip in pounds-force. Compared with Jamar dynamometer, the Saehan hydraulic dynamometer is also a valid and trusty instrument [28].

BSCL and healthy subjects were kept in a seated position and received verbal instructions for completing the assessment. We used the mean of four grip trials as compared to either a single grip trial or the highest reading of four trials. The predicted values for grip strength were measured according to the Brazilian population previously described by Caporrino et al. [29]. Measurements of maximal respiratory pressures and peripheral muscle strength were performed by the same investigator.

### Statistical analysis

The data were analyzed using *GraphPad Prism* software, version 6.0. The Shapiro–Wilk test was used to analyze normality data distribution. Continuous and normally distributed variables are expressed as mean ± standard deviation (SD). Proportions are presented as number (%) for categorical variables. Parametric comparisons between the groups were performed by unpaired T-test. The intragroup analysis was performed by repeated measures ANOVA followed by a Bonferroni post hoc test. To evaluate the correlation between MIP and peripheral muscle strength, Pearson (parametric variables) and Spearman (non-parametric variables) correlation coefficients were used. Associations between categorical variables were analyzed by the chi-square test. Statistical significance was set at *p* <  0.05.

## Results

Eleven BSCL subjects were included in this study. Clinical, metabolic, and genetic data were obtained (Table 1). We found that the majority of BSCL subjects were Type 2, presenting a specific mutation in the BSCL2 gene (*325dupA*). Only one patient was Type 1 (case 1), presenting the *A712T* mutation in the AGPAT2 gene. There were no significant differences between BSCL and control subjects for gender, age, height, weight, and BMI (Table 2). Table 3 summarizes the weekly frequency of physical activities according to IPAQ. We found that 63.6% (*n* = 7) of the BSCL group were classified as irregularly active B to very active, while 36.4% (*n* = 3) were classified as sedentary. On the other hand, 36.5% of the control group were classified as irregularly active B to very active, while 63.6% were classified as sedentary. These data indicate that in 2015 the BSCL group were more active than the healthy volunteers.

**Table 2:** Physiological and metabolic data (mean ± SD) of BSCL subjects and healthy volunteers (Control)

(*n* = 11)	BSCL	Control	*p*
Age (years)	31.45 ± 8.04	30.91 ± 8.13	0.8759
Height (m)	1.65 ± 0.09	1.63 ± 0.08	0.6531
Weight (kg)	59.3 ± 13.45	64.85 ± 14.80	0.3680
BMI (kg/m^2^)	21.56 ± 3.08	24.18 ± 4.41	0.1223
Diabetes Mellitus (n/%)	11 (100)	0 (0)	< 0.0001
Fasting glycemia (mg/dL)	268 ± 124	NA	–
Serum triglycerides (mg/dL)	173 ± 94.84	NA	–
Total cholesterol (mg/dL)	151.3 ± 63.65	NA	–

*p* values were based on independent t-tests. For categorical variables, the *p* value was calculated using the chi-square test

*BMI* body mass index, *NA* Not available

**Table 3:** Physical activity data of BSCL subjects and healthy volunteers (Control)

Classification^a^	BSCL (n/%)	Control (n/%)
Sedentary	4 (36.36)	7 (63.63)
Irregularly active B	1 (9.09)	1 (9.09)
Irregularly active A	1 (9.09)	2 (18.18)
Active	4 (36.36)	1 (9.09)
Very active	1 (9.09)	0 (0.00)
Total	11 (100)	11 (100)

^a^According to IPAQ [17–19]

**Fig. 1:**
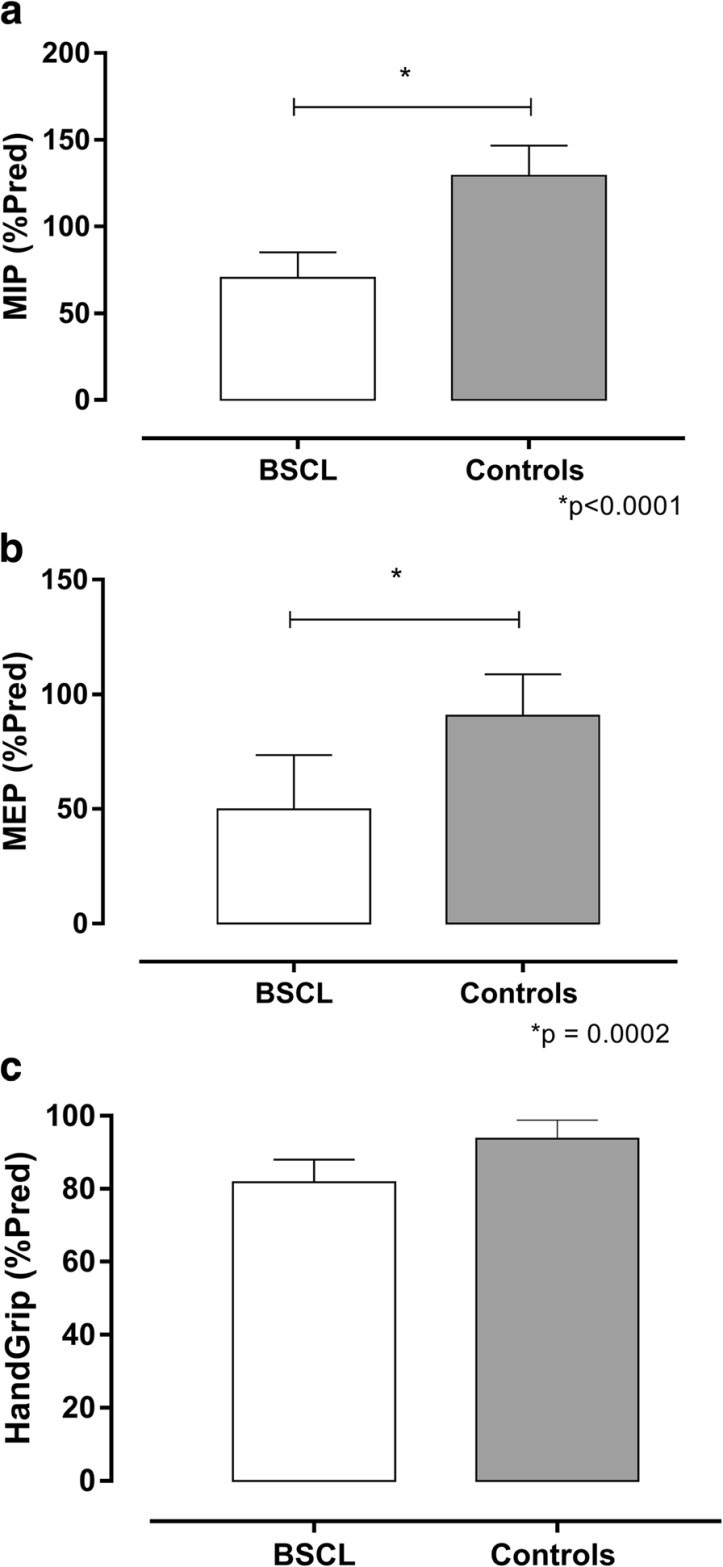
Maximum Inspiratory Pressure (MIP), Maximum Expiratory Pressure (MEP) and peripheral muscle strength (PMS) values of the 11 BSCL patients and healthy subjects at the first year. **a** MIP, (**b**) MEP, and (**c**) PMS indices are expressed as % of predicted values. The results are represented as the mean ± SD. The differences were considered statistically significant when *p < 0.05 using unpaired Student’s t-test

The BSCL group showed lower MIP and MEP pressures but no changes in handgrip strength. Although BSCL case 8 was a smoker, he presented a similar profile of these variables compared to all BSCL subjects. For this purpose, we decided to maintain case 8 in this research. Controls have shown significantly higher values for MIP and MEP in comparison to BSCL individuals in the first year. No significant difference between the groups was found for grip strength (PMS) (Table 4; Fig. 1).

**Table 4:** MIP, MEP, and PMS measurements (mean ± SD and [95% confidence interval]) in BSCL subjects and healthy volunteers (Control)

(*n* = 11)	MIP (predicted %)	MEP (predicted %)	PMS (predicted %)
BSCL subjects	70.24 ± 14.89	49.78 ± 23.81	81.77 ± 20.71
	[60.24–80.24]	[33.79–65.78]	[67.85–95.68]
Controls	129.3 ± 17.56	90.83 ± 17.83	93.76 ± 16.84
	[117.5–141.5]	[78.85–102.8]	[82.45–105.1]
*p*	*< 0.0001*	*0.0002*	0.1517

*p* values were based on independent unpaired t-tests

**Fig. 2:**
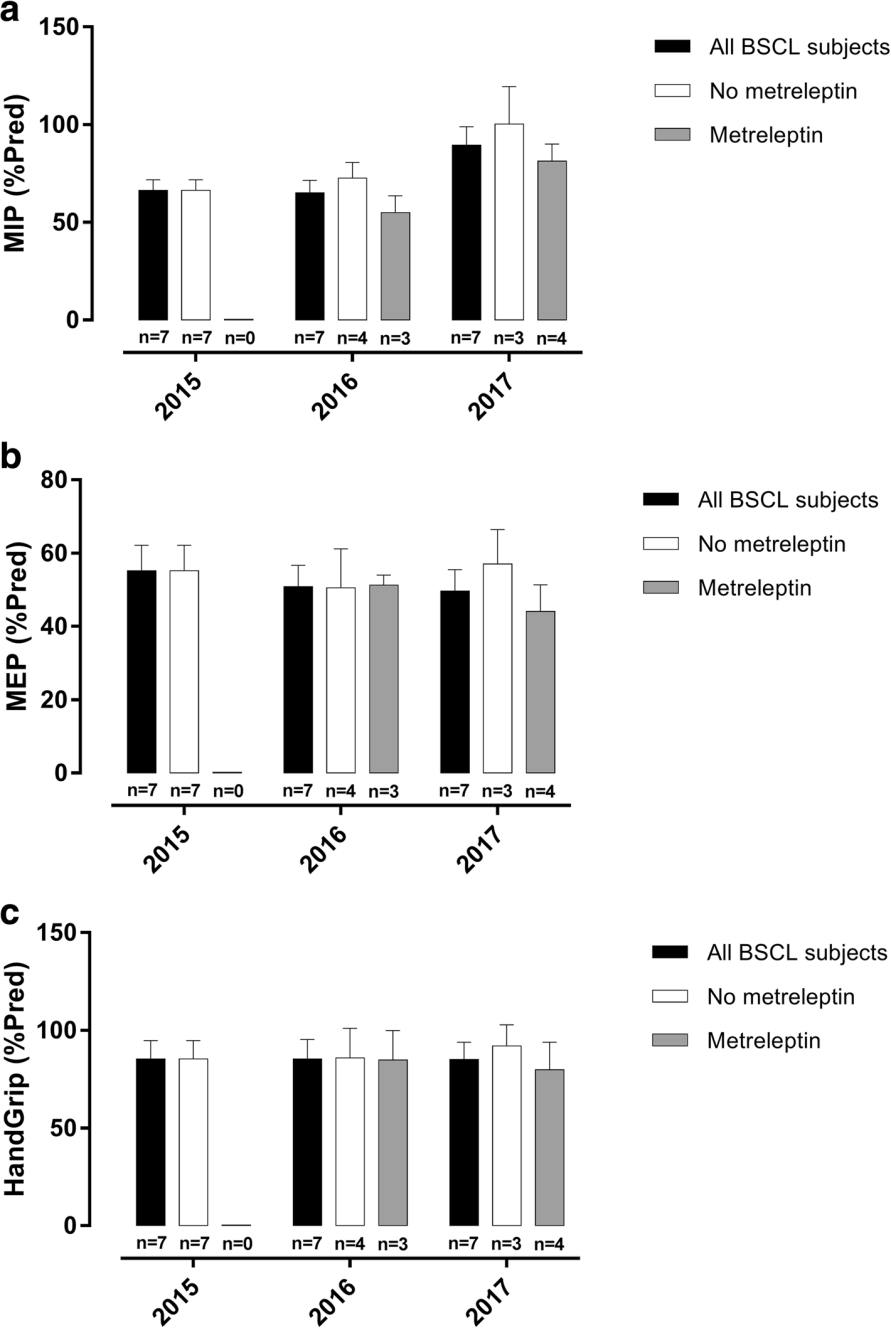
Maximum Inspiratory Pressure (MIP), Maximum Expiratory Pressure (MEP), and peripheral muscle strength (PMS) values of all BSCL subjects and after stratification according to the use of metreleptin from 2015 to 2017. **a** MIP, (**b**) MEP, and (**c**) PMS indices are expressed as % of predicted values. The results are represented as the mean ± SD. For the analysis of all BSCL subjects, the differences were considered statistically significant when *p < 0.05 using repeated measures ANOVA. For the analysis after stratification according to the use of metreleptin, the differences were considered statistically significant when *p < 0.05 using repeated measures ANOVA for the group without metreleptin and using unpaired Student’s t-test for the group with metreleptin

**Fig. 3:**
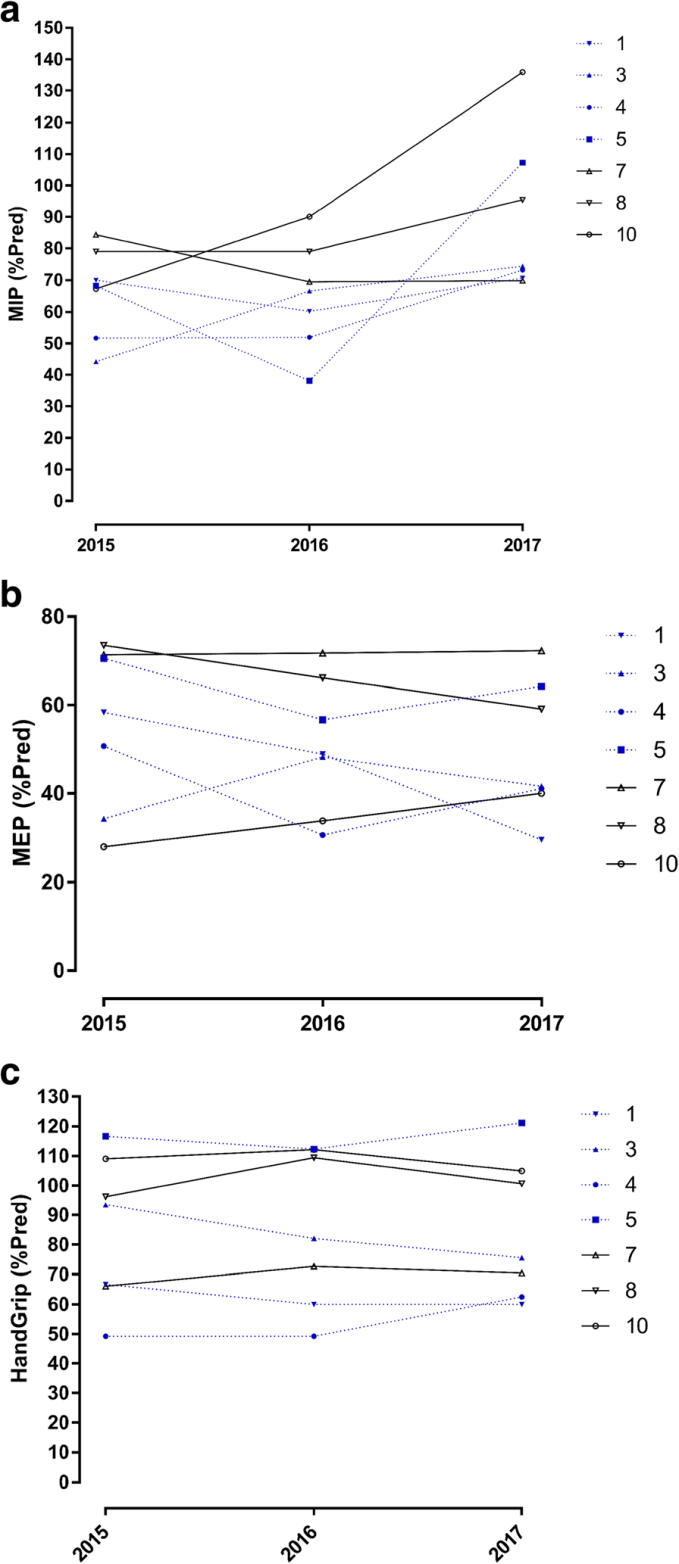
Maximum Inspiratory Pressure (MIP), Maximum Expiratory Pressure (MEP), and peripheral muscle strength (PMS) values for each BSCL subject at 2015, 2016, and 2017. **a** MIP, (**b**) MEP, and (**c**) PMS indices are expressed as % of predicted values

Additionally, even if MIP, MEP, and peripheral muscle strength gradually increased after the first investigation, these variations were not statistically significant among the three evaluations, revealing that BSCL subjects presented maintenance of respiratory dysfunction over the years (Table 5; Fig. 2). To better understand the role of metreleptin in the respiratory and peripheral muscle strength of BSCL subjects, we divided the BSCL subjects according to the use of metreleptin (Table 6 and Fig. 2). The data revealed that the metreleptin replacement did not result in an improvement of MIP, MEP, and peripheral muscle in BSCL subjects. Moreover, we also examined the results of these tests for each BSCL patient separately. As shown in Fig. 3, metreleptin did not improve MIP, MEP, and peripheral muscle strength, since a similar profile was observed for subjects without (7, 8, and 10 - solid black lines) and with (1, 3, 4, and 5 - dashed blue lines) metreleptin replacement.

**Table 5:** MIP, MEP, and PMS measurements (mean ± SD and [95% confidence interval]) in all BSCL subjects after three evaluations

(*n* = 7)	2015	2016	2017	*P*
MIP (predicted %)				
All BSCL subjects	66.34 ± 14.2	65.04 ± 17.14	89.5 ± 24.96	0.0580
	[53.21–79.47]	[49.18–80.9]	[66.41–112.6]	
MEP (predicted %)				
All BSCL subjects	55.24 ± 18.42	50.89 ± 15.35	49.68 ± 15.52	0.4573
	[38.20–72.28]	[36.69–65.09]	[35.33–64.03]	
PMS (predicted %)				
All BSCL subjects	85.34 ± 25	85.41 ± 26.28	85.05 ± 23.73	0.9914
	[62.22–108.5]	[61.11–109.7]	[63.1–107]	

*p* values were based on repeated measures ANOVA

**Table 6:** MIP, MEP, and peripheral muscle strength measurements (mean ± SD and [95% confidence interval]) in BSCL subjects without and with metreleptin replacement

(*n* = 7)	2015 (n/%)	2016 (n/%)	2017 (n/%)	*P*
MIP (predicted %)				
No Metreleptin	7 (100)	4 (57.2)	3 (42.8)	–
	70.24 ± 14.89	72.62 ± 16.14	100.4 ± 33.27	^b^0.0719
	[60.24–80.24]	[46.93–98.30]	[17.71–183.00]	–
Metreleptin	0 (0)	3 (42.8)	4 (57.2)	–
	–	54.93 ± 14.93	81.35 ± 17.36	^a^0.0892
	–	[17.84–92.03]	[53.73–109]	
*p*^a^	–	0.7524	0.3644	
MEP (predicted %)				
No Metreleptin	7 (100)	4 (57.2)	3 (42.8)	–
	55.24 ± 18.42	50.56 ± 21.34	57.1 ± 16.19	^b^0.8881
	[38.2–72.28]	[16.6–84.52]	[16.87–97.33]	–
Metreleptin	0 (0)	3 (42.8)	4 (57.2)	–
	–	51.31 ± 4.67	44.11 ± 14.51	^a^0.4541
	–	[39.69–62.93]	[21.03–67.19]	–
*p*^a^	–	0.9556	0.3142	–
PMS (predicted %)				
No Metreleptin	7 (100)	4 (57.2)	3 (42.8)	–
	85.34 ± 25	85.87 ± 30.34	92.03 ± 18.78	^b^0.9270
	[62.22–108.5]	[37.6–134.1]	[45.38–138.7]	–
Metreleptin	0 (0)	3 (42.8)	4 (57.2)	–
	–	84.8 ± 26.27	79.81 ± 28.39	^a^0.8219
	–	[19.53–150.1]	[34.63–125]	–
*p*^a^	–	0.9632	0.5504	–

^a^*p* values were based on independent unpaired t-tests

^b^*p* values were based on repeated measures ANOVA for the group without metreleptin

**Fig. 4:**
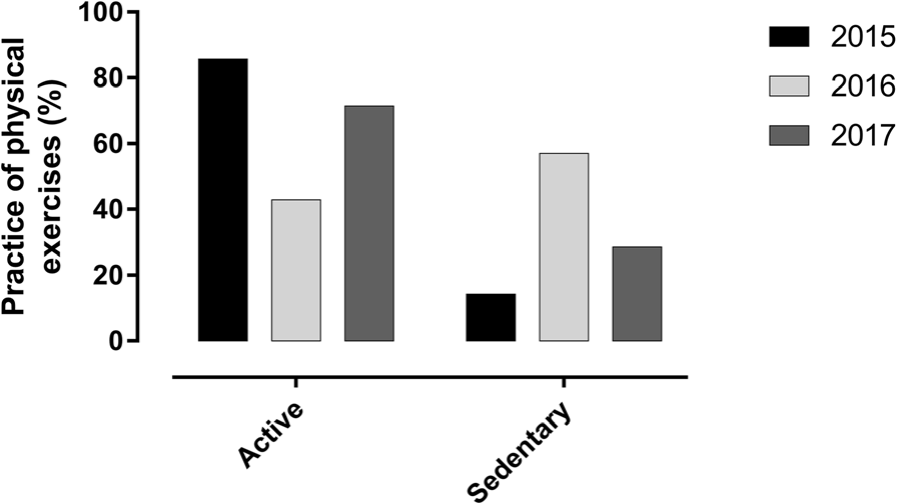
Profile of physical exercise practices of BSCL subjects from 2015 to 2017 according to IPAQ classification. The results are represented as %. The differences were considered statistically significant when *p < 0.05 using two-way ANOVA with multiple comparisons (Bonferroni post hoc test)

Afterward, we investigated the weekly frequency of physical exercise practices among the three evaluations. We observed that 42.8% (*n* = 3) of BSCL subjects presented the same profile during the 2 years of investigation (Additional file 1: Table S1). Instead, 57.2% (*n* = 4) showed changes in their physical activity profile, varying from active to sedentary, and irregularly active to sedentary. Even if we consider all BSCL subjects with one of the active profiles in the same group, our data confirm that the BSCL group were more active in 2015 and 2017 (Fig. 4).

In addition, we found that grip strength and MIP were not correlated in these subjects (Additional file 2: Figure S1). We also investigated whether the respiratory and peripheral muscle strength were correlated with the metabolic variables assessed in this study. We found that the tests used to study the respiratory and peripheral strength did not present an association with age, BMI, glucose, triglycerides, and total cholesterol (Additional file 1: Table S2).

## Discussion

In this study, we showed a decrease in the maximum static respiratory pressures of subjects with BSCL. This condition was not modified after 2 years as indicated by the maintenance of the low levels of MIP and MEP observed from 2015 to 2017. No changes were observed in the peripheral muscle strength of those subjects. To our knowledge, this is the first study that verified respiratory and peripheral muscle strength in this population.

Several studies showed a decrease in static respiratory pressures in patients with metabolic, renal, and cardiovascular complications [30–33], findings that were also described in BSCL patients [3–5, 16, 34]. All BSCL subjects from our research had diabetes, as shown in Table 1, which is in accordance with the previously published data from our population [3, 14].

There is evidence that the lung is a target organ in diabetes mellitus and pulmonary complications, as a respiratory muscle strength weakness was found in diabetic patients, correlating with a poor metabolic control [13]. Some mechanisms were proposed to explain the role of diabetes mellitus in pulmonary function. An association between autonomic neuropathy related to the respiratory muscles and lung impairment was previously identified [35]. Diabetic autonomic neuropathy is a complication of diabetes mellitus that has been associated with cardiovascular and respiratory dysfunctions in several studies [36–41]. Concerning BSCL, Faria et al. [5] previously showed abnormal autonomic modulation in BSCL patients of our cohort by heart rate variability (HRV) analysis, and this was independent of the presence of metabolic and hemodynamic disturbances. Atypical autonomic modulation was also recently confirmed in BSCL patients from Ceará, a Northeast state located near our patients’ state [16].

Furthermore, subjects presenting type 2 diabetes mellitus with autonomic neuropathy also showed reduced muscle strength and less HR variability and the researchers found a positive correlation between MIP and HRV [12]. Together, these results suggest that BSCL subjects could present respiratory muscle weakness. The results of the present study revealed a reduction of MIP and MEP in BSCL subjects that persisted for over two years of evaluation.

In both studies, almost all BSCL patients presented the *325dupA* mutation in the BSCL2 gene that encodes Seipin. BSCL type 2 is the most severe form of this lipodystrophy, and the propensity to premature death is higher in these patients [3, 7], which could explain the low average age found for this BSCL group (31.4 ± 8.0 years). There are many Seipin-related motor neuropathies, named Seipinopathy. Lipodystrophy occurs due to Seipin loss-of-function, and the nervous system is slightly affected when compared with Seipinopathies, which occur due to Seipin gain-of-function [1, 42].

Seipin also plays an important role in regulating excitatory synaptic transmission [43]. We hypothesize that Seipin deficiency could affect the contraction of inspiratory and expiratory musculatures due to an imbalance of excitatory and inhibitory synaptic transmission. Sympathetic activation can be induced by inspiratory muscle fatigue, resulting in increased cardiac debt and arterial pressure and, consequently, leading to a reduction in arterial blood flow to the lower limbs. These findings indicate an interaction between ventilatory muscle activity and cardiovascular control mechanisms, which could potentiate previous abnormal autonomic control [12, 44, 45]. However, since these explanations were most pronounced in diabetic patients presenting autonomic neuropathies [12], new investigations are needed to address the role of Seipin in the regulation of autonomic neuropathies and its relationship with respiratory muscle strength.

Seipin is also implicated in ER stress, and this phenomenon was widely studied in liver, adipose tissue, pancreatic islets, and mammary gland alveolar epithelial cells [46–49]. However, there is a lack of information about ER stress and Seipin’s role in skeletal muscle pathophysiology. While Seipinopathy was shown to be related to ER stress, Lipodystrophy was not associated with ER stress in non-adipogenic cells. In Silver Syndrome, a type of Seipinopathy, a weakness of upper body strength was observed, indicating that Seipin gain of function can affect the upper musculature [50]. In fact, *Bscl2* is robustly expressed in the XI cranial nerve, the main source of efferent motor fibers to the muscle groups of the shoulder and neck [50]. We suppose that there is a relationship between ER stress and Seipin in respiratory skeletal muscles that may not be per se associated with Seipinopathy, since our BSCL type 2 patients (*325dupA* mutation) did not present any signs related to neurological disturbances that were described for Seipinopathy.

The diaphragm is the most important skeletal muscle for respiratory function and, together with other respiratory muscles, acts as a respiratory pump, generating during inspiration the tidal volume necessary for the proper alveolar ventilation. Hyperglycemia can lead to loss of diaphragm muscle strength due to increased production of reactive oxygen species (ROS), which can damage contractile proteins, such as troponin T [51]. In addition, it is possible that insulin resistance, caused by intramuscular triglyceride accumulation, especially in mitochondria, generates negative adaptations in the diaphragm morphology and function, leading to the development of respiratory muscle weakness [52]. In this research all of the BSCL subjects presented hypertriglyceridemia and diabetes mellitus, indicating that these comorbidities may be associated with inspiratory muscle weakness found in these subjects.

Although leptin has been shown to be related to diabetic complications, no data from exploring its role in respiratory function are available. Since high levels of leptin were associated with autonomic neuropathy in diabetic patients [53–55] and these patients presented decreased muscle strength [12], we hypothesized that a relationship could exist between leptin and respiratory function. We observed a tendency to an increase in MIP and MEP after leptin replacement when all BSCL subjects were examined together (Fig. 2; Table 4). However, since only four BSCL subjects of our study started the metreleptin replacement, we were not able to conclude that administration of metreleptin can result in an improvement of respiratory muscle strength because BSCL subjects without metreleptin replacement also presented a slight increase in MIP and MEP when compared to those with metreleptin replacement (Figs. 2 and 3; Table 5).

The association between respiratory and peripheral muscle strength was previously studied in the literature. It was postulated that MIP could equally reflect the peripheral muscle strength and that the reverse is similarly true [56, 57]. In this context, we also evaluated the occurrence of peripheral muscle strength in our BSCL subjects, and the occurrence of a correlation between MIP and peripheral muscle strength was verified. In our study, we did not find peripheral muscle weakness in all BSCL subjects evaluated. These data reveal a difference between Lipodystrophy and Seipinopathy, since in the latter the weakness of upper limbs was previously described for Silver Syndrome [50].

Moreover, our peripheral muscle strength data are opposite to those obtained by Garg and co-workers [58]. The researchers observed reduced muscle strength of quadriceps femoris in 3 African BSCL patients. However, the genotyping of all patients evaluated in that study was not performed and no suppositions concerning the mutated gene and the specific BSCL mutation with the weakness of quadriceps muscle strength can be done. We also could not compare our results of MIP, MEP, and peripheral muscle strength according to the type of BSCL mutation since we had only 1 BSCL type 1 patient in our study. The one type 1 BSCL patient (case 1) presented maintenance of MIP during the 2 years of follow-up. Nonetheless, MEP and handgrip strength were reduced at follow-up, remaining below 70% of the predicted values. Weakness of lower muscle strength was also shown in patients with metabolic syndrome, especially in diabetic patients with or without polyneuropathy [59, 60].

To better elucidate the morphology of skeletal muscle from BSCL patients, Garg and co-workers also evaluated biopsy samples from quadriceps femoris and found an increase in glycolytic muscle fibers and a reduction in oxidative muscle fibers compared to healthy women. They postulated that insulin resistance found in those BSCL patients could be associated with the increase in glycolytic muscle fibers [58]. They proposed that the increased muscularity in BSCL is due to muscle hyperplasia and is not associated with increased muscle strength.

An inefficient metabolic control can contribute to a reduction in resistance to fatigue, which can be explained by the morphophysiological changes of respiratory and peripheral muscles, resulting in microvascular perturbations related to insulin resistance that compromises muscle strength and endurance [13, 58]. Nevertheless, we did not find any correlation between the respiratory and peripheral strength with the metabolic variables studied in our research. We suggest that the difference observed in our study, compared with Garg [58], can be explained by the specific characteristics of upper and lower limbs. The latter are more resistant to fatigue, mainly the quadriceps, when compared with upper limbs. In the case of BSCL patients from Garg’s research [58], the increased levels of glycolytic fibers resulted in a reduction in muscle strength probably due to the commitment of the muscle performance. Currently, no data are available about the morphology of upper muscles from BSCL patients. In addition, the lower limbs may be more affected by the disuse. Domestic and personal care activities may prevent muscle weakness in the upper limbs, which are more affected in the advanced stages of metabolic comorbidities [61–64].

It has been shown that inspiratory muscle training could improve inspiratory muscle function with no consequences in functional capacity or autonomic modulation [65, 66]. Inspiratory muscle training applied for patients with metabolic syndrome has been essential to prevent and/or treat respiratory muscle weakness [65]. The lack of physical exercise is a central mechanism in the development of diabetes mellitus and can contribute to reduced muscle strength. We believe that it is essential to verify MIP and MEP in healthy diabetic subjects to compare with our data found in diabetic BSCL patients. In addition, since metabolic disturbances, including diabetes, are a marked commitment found in BSCL patients, new physical interventions are required to improve the metabolic conditions and respiratory muscle strength of these patients.

## Conclusions

We found an association between BSCL and decreased respiratory muscle strength during the 2 years of evaluation, suggesting that this lipodystrophy may be related to respiratory muscle weakness, which was not recovered by metreleptin treatment. The accurate molecular and pathophysiological mechanisms to explain these results require further investigation to reveal the impact of metabolic control and the practice of physical activity on respiratory muscle strength of Berardinelli-Seip Congenital Lipodystrophy subjects.

## Study limitations

The measurement of respiratory muscle strength by maximum static respiratory pressure tests may be considered a study limitation due to its volitional characteristic. Non-volitional tests, such as the magnetic and electrical stimulation of phrenic nerve, are highly specific. However, the application of non-volitional tests presents high cost and is less applicable in the clinical practice. Furthermore, it is important to increase the number of BSCL subjects with type 1 BSCL and include the younger type 2 BSCL subjects to better understand the role of 1-AGPAT2 and Seipin on respiratory and peripheral muscle strength. The increase of BSCL subjects will also allow better description of the difference between Lipodystrophy and Seipinopathy regarding the weakness of upper limbs.

## Additional files

Additional file 1: **Table S1.** Physical activity data of BSCL subjects after three evaluations. **Table S2.** Correlations between respiratory and peripheral strength tests and metabolic parameters of BSCL subjects. (DOCX 18 kb)

Additional file 2: **Figure S1.** Correlation between MIP and peripheral muscle strength (PMS) values of BSCL subjects. MIP indices at 2015, 2016, and 2017 were used. r values of a Pearson correlation coefficient and *p* values are included. (TIF 345 kb)

## Abbreviations

AGPAT: Acylglycerol-3-phosphate O-acyltransferase
ANOVA: Analysis of variance
*ASPOSBERN*: Association of Parents and People with Berardinelli Syndrome of *Rio Grande do Norte* (*Associação dos Pais e Pessoas com a Síndrome de Berardinelli do Estado do Rio Grande do Norte*)
ATS/ERS: *American Thoracic Society* and *European Respiratory Society*
BMI: Body mass index
BSCL: Berardinelli-Seip Congenital Generalized Lipodystrophy
CI: Confidence interval
ER: Endoplasmic reticulum
FACISA: *Faculdade de Ciências da Saúde do Trairi*
HRV: Heart rate variability
MEP: Maximum Expiratory Pressure
MIP: Maximum Inspiratory Pressure
PMS: Peripheral Muscle Strength
PMW: Peripheral Muscle Weakness
RMS: Respiratory Muscle Strength
RMW: Respiratory Muscle Weakness
RN: Rio Grande do Norte
UFRN: Universidade Federal do Rio Grande do Norte
