# Supplementary material for: Impairment of respiratory muscle strength in Berardinelli-Seip congenital lipodystrophy subjects
Source: Respir Res. 2018 Sep 12;19:173. doi: 10.1186/s12931-018-0879-8 (PMC6134719; doi:10.1186/s12931-018-0879-8)
Supplement: Supplementary file 1 — Table S1. Physical activity data of BSCL subjects after three evaluations. Table S2. Correlations between respiratory and peripheral strength tests and metabolic parameters of BSCL subjects. (DOCX 18 kb) [file 12931_2018_879_MOESM1_ESM.docx]

**RERE-D-18-00227**

**Impairment of Respiratory Muscle Strength in Berardinelli-Seip Congenital Lipodystrophy Subjects**

Jorge Luiz Dantas de Medeiros^a^, Bruno Carneiro Bezerra^b^, Thiago Anderson Brito de Araújo^c^, Aquiles Sales Craveiro Sarmento^d^, Lázaro Batista de Azevedo Medeiros^e^, Lucien Peroni Gualdi^f^, Maria do Socorro Luna Cruz^g^, Thaiza Teixeira Xavier Nobre^h^, Josivan Gomes Lima^i^, Julliane Tamara Araújo de Melo Campos^j^*

______________________________________________________________

**Supplementary Information**

| Additional file 1: Table S1. Physical activity data of BSCL subjects after three evaluations. | | | |
| --- | --- | --- | --- |
| **Case** | **2015 classification^#^** | **2016 classification^#^** | **2017 classification^#^** |
| **1** | Active | Sedentary* | Sedentary |
| **3** | Irregularly active A | Sedentary* | Irregularly active A |
| **4** | Active | Active | Irregularly active B* |
| **5** | Sedentary | Sedentary* | Sedentary |
| **7** | Active | Active | Active |
| **8** | Very active | Very active | Very active |
| **10** | Irregularly active B | Sedentary | Irregularly active B |
| ^#^According to IPAQ [17–19]. ^*^ Indicates the beginning of metreleptin replacement. | | | |

| Additional file 1: Table S2. Correlations between respiratory and peripheral strength tests and metabolic parameters of BSCL subjects. | | | | |
| --- | --- | --- | --- | --- |
| **(n = 11)** |  | **MIP (predicted %)** | **MEP (predicted %)** | **PMS (predicted %)** |
| **Age** | r  p | 0.226  0.504 | 0.134  0.695 | 0.235  0.486 |
| **BMI** | r  p | 0.118  0.729 | 0.082  0.810 | 0.599  0.052 |
| **Glucose** | r  p | -0.207  0.565 | -0.307  0.388 | 0.019  0.958 |
| **Triglycerides** | r  p | 0.150  0.678 | -0.519  0.124 | 0.353  0.318 |
| **Total cholesterol** | r  p | 0.067  0.857 | -0.602  0.070 | 0.559  0.097 |
| Test for age, BMI, glucose and triglycerides variables: Pearson’s correlation. Test for total cholesterol variable: Spearman’s correlation. BMI: Body mass index. | | | | |
