# Supplementary figures and images for: Impairment of respiratory muscle strength in Berardinelli-Seip congenital lipodystrophy subjects
Source: Respir Res. 2018 Sep 12;19:173. doi: 10.1186/s12931-018-0879-8 (PMC6134719; doi:10.1186/s12931-018-0879-8)

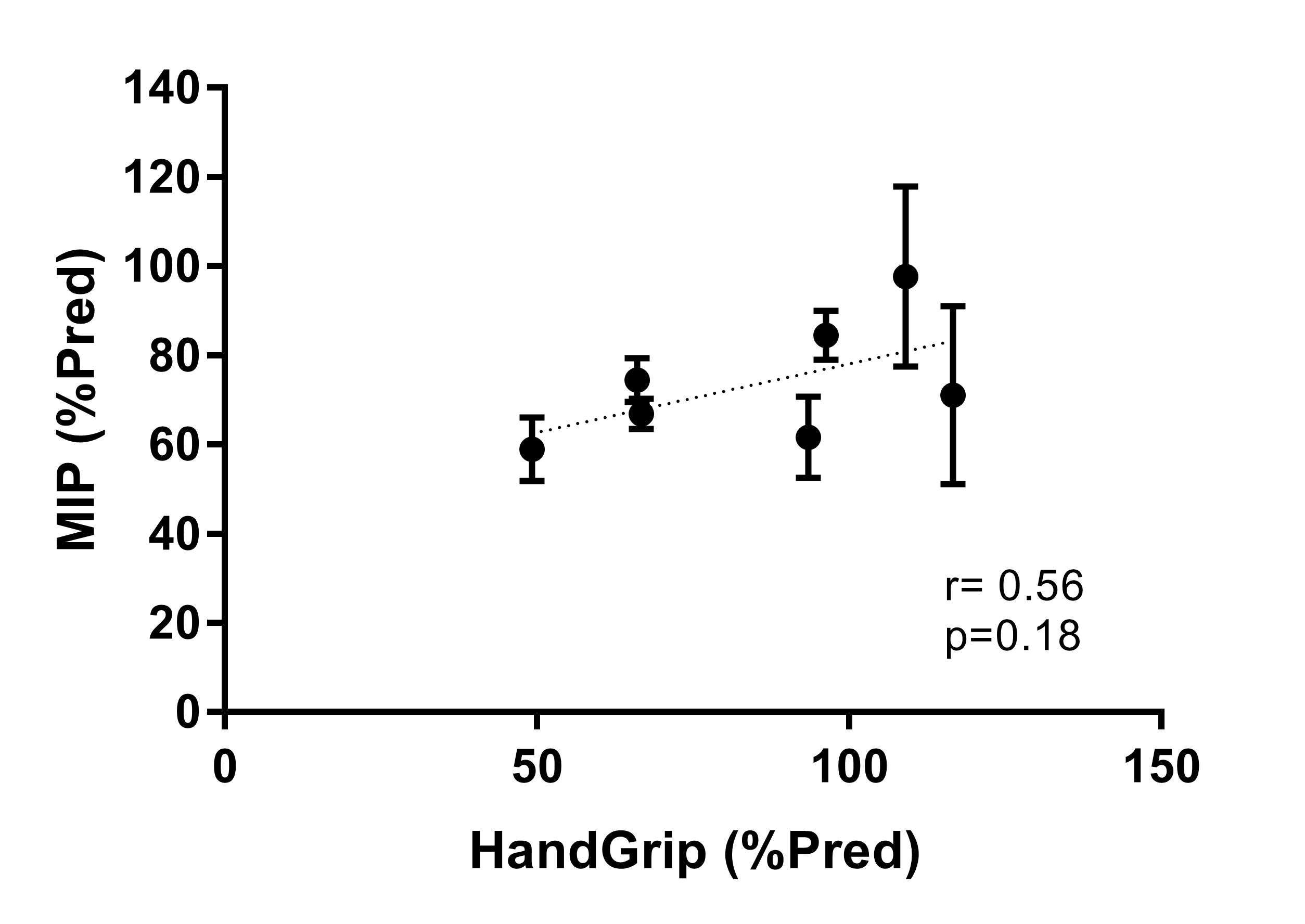

Supplement: Supplementary file 2 — Figure S1. Correlation between MIP and peripheral muscle strength (PMS) values of BSCL subjects. MIP indices at 2015, 2016, and 2017 were used. r values of a Pearson correlation coefficient and p values are included. (TIF 345 kb) [file 12931_2018_879_MOESM2_ESM.tif]
